# mTORC1 plays an important role in osteoblastic regulation of B-lymphopoiesis

**DOI:** 10.1038/s41598-018-32858-5

**Published:** 2018-09-28

**Authors:** Sally K. Martin, Stephen Fitter, Nadia El Khawanky, Randall H. Grose, Carl R. Walkley, Louise E. Purton, Markus A. Ruegg, Michael N. Hall, Stan Gronthos, Andrew C. W. Zannettino

**Affiliations:** 1 Myeloma Research Laboratory, Adelaide Medical School, Faculty of Health and Medical Sciences, University of Adelaide, Adelaide, Australia; 2 The South Australian Health and Medical Research Institute, Adelaide, Australia; 3 School of Medicine, Faculty of Health and Medical Sciences, University of Adelaide, Adelaide, Australia; 4 Department of Hematology and Oncology, Faculty of Medicine, University of Freiburg, Freiburg, Germany; 5 Stem Cell Regulation Unit, St Vincent’s Institute of Medical Research, Melbourne, Australia; 6 Biozentrum, University of Basel, Basel, Switzerland; 7 Mesenchymal Stem Cell Laboratory, Adelaide Medical School, Faculty of Health and Medical Sciences, University of Adelaide, Adelaide, Australia

## Abstract

Skeletal osteoblasts are important regulators of B-lymphopoiesis, serving as a rich source of factors such as CXCL12 and IL-7 which are crucial for B-cell development. Recent studies from our laboratory and others have shown that deletion of *Rptor*, a unique component of the mTORC1 nutrient-sensing complex, early in the osteoblast lineage development results in defective bone development in mice. In this study, we now demonstrate that mTORC1 signalling in pre-osteoblasts is required for normal B-lymphocyte development in mice. Targeted deletion of *Rptor* in osterix-expressing pre-osteoblasts (*Rptor*_*ob*_^−/−^) leads to a significant reduction in the number of B-cells in the bone marrow, peripheral blood and spleen at 4 and 12 weeks of age. *Rptor*_*ob*_^−/−^ mice also exhibit a significant reduction in pre-B and immature B-cells in the BM, indicative of a block in B-cell development from the pro-B to pre-B cell stage. Circulating levels of IL-7 and CXCL12 are also significantly reduced in *Rptor*_*ob*_^−/−^ mice. Importantly, whilst *Rptor*-deficient osteoblasts are unable to support HSC differentiation to B-cells in co-culture, this can be rescued by the addition of exogenous IL-7 and CXCL12. Collectively, these findings demonstrate that mTORC1 plays an important role in extrinsic osteoblastic regulation of B-cell development.

## Introduction

From late embryogenesis onwards, the bone marrow (BM) is the primary site of haematopoietic stem cell (HSC) self-renewal and differentiation. The process of HSC differentiation into the various blood cell types is tightly regulated by the BM microenvironment, which consists of stromal cells (osteoblasts, endothelial cells, macrophages, fibroblasts, adipocytes), extracellular matrix and soluble regulatory factors. In recent years, researchers have investigated the specific contribution of individual BM cell types to the HSC niche and so far, the greatest importance has been attributed to bone-forming osteoblasts and the vasculature (reviewed in^1,2^).

Anatomically, quiescent HSCs reside close to the endosteal surface of the trabecular bone in an HSC reservoir that can be mobilised in response to physiological demand or tissue injury. Osteoblast cells secrete several factors (e.g. angiopoietin, BMP4, thrombopoietin, CXCL12) that are important for HSC self-renewal, survival and maintenance. The selective ablation of osteoblasts *in vivo* leads to a dramatic reduction in BM cellularity and haematopoiesis^3^ and conversely, when the number of osteoblasts are experimentally increased *in vivo*, a concomitant increase in the BM HSC pool size, but not the hematopoietic cell pool size, is observed^4,5^.

Beyond its support for HSCs, osteoblasts are also important for the support of differentiated haematopoietic cells such as B-cells. Osteoblasts have been shown to support all stages of B-cell development *in vitro*, from the first transition of HSCs to the common lymphoid progenitor to the generation of mature IgM^+^ B-cells^6^. When endosteal osteoblasts are conditionally ablated *in vivo*, B-cell lymphopoiesis is abruptly and drastically reduced^3,6^.

The BM microenvironment contains distinct niches for each stage of B-lymphocyte development. B-lymphocytes originate from HSCs through a common lymphoid progenitor. The earliest identifiable B-cell precursor in the BM are prepro-B cells, which sequentially differentiate into pro-B cells, pre-B cells and immature B-cells. These immature B-cells then migrate into the periphery, where final maturation occurs in the spleen^7–12^. Within the BM microenvironment, CXCL12 and interleukin (IL)-7, two factors that are important in B-cell development^4,6,13^, are expressed by distinct stromal cell populations. Importantly, prepro-B cells are found in close proximity to CXCL12-expressing cells, whereas the more differentiated pro-B cells are found in contact with IL-7-expressing cells^14^. These findings suggest that as B-cell precursors differentiate, they migrate between discrete populations of supporting stromal cells in the BM microenvironment.

Bone cells arise from multipotent mesenchymal stem cells which, in response to specific cues, undergo sequential differentiation into osteoprogenitors, pre-osteoblasts, mature osteoblasts and terminally-differentiated osteocytes. Conditional deletion of CXCL12 in osteoprogenitors, but not mature osteoblasts, results in a marked loss of early B-cell progenitors^15^, and ablation of the G_s_-alpha subunit of the parathyroid receptor in osteoprogenitors, but not mature osteoblasts, results in impaired B-lymphopoiesis^16,17^. This suggests that osteoprogenitors, and not mature osteoblasts, play a crucial role in B-lymphopoiesis.

Most cellular processes that govern growth require activation by anabolic signals. Mammalian target of rapamycin (mTOR) is an evolutionarily conserved kinase that functions in two structurally and functionally distinct multiprotein complexes, termed mTORC1 and mTORC2, which are defined by the unique adaptor proteins raptor and rictor, respectively. Activated by major intracellular and extracellular stimuli, mTORC1 controls protein synthesis, ribosome biogenesis and nutrient transport through the activation of downstream effectors p70S6K and 4-EBP1^18,19^. Notably, recent studies from our laboratory^20^ and others^21,22^ have shown that conditional deletion of mTORC1 in pre-osteoblast cells (*Rptor*_*ob*_^−/−^) results in a marked reduction in pre- and post-natal bone accrual, reduced limb length and increased skeletal fragility due to a reduction in osteoblast function. In light of the prominent skeletal phenotype of *Rptor*_*ob*_^−/−^ mice and the critical role that osteoblasts play in B-cell development, we hypothesised that osteoblastic mTORC1 may also play an important role in regulating B-lymphocyte development *in vivo*. To investigate this, we examined B-lymphopoiesis in our *Rptor*_*ob*_^−/−^ mice and found a significant reduction in the number of B-lymphocytes in the bone marrow, spleen and peripheral blood. Furthermore, we found that the mTORC1-dependent regulation of B-lymphopoiesis by osteoblastic cells is mediated by CXCL12 and IL-7.

## Results

### Conditional Ablation of *Rptor* in Osteoblast Precursors leads to Impaired B Lymphopoiesis

*Rptor*, a unique and essential component of mTORC1, was ablated in osteoblast precursor cells using transgenic mice in which Cre recombinase is driven by the osterix promoter, a transcription factor expressed early in osteoblastogenesis (*Osx1-GFP::Cre*)^23^. These mice were mated with mice harbouring loxP sites flanking exon 6 of the *Rptor* gene (*Rptor*^*fl/fl*^)^24^ and a floxed stop *R26-stop-EYFP* reporter^25^ to produce heterozygous (*eYFP-Rptor*_*ob*_^+/−^) and homozygous (*eYFP-Rptor*_*ob*_^−/−^) *Rptor* knockout mice^20^. In light of the distinct skeletal phenotype of *Osx1-GFP::Cre* mice^20,26–28^, *eYFP-Osx:Cre* transgenic mice (as opposed to wild type littermates) were used as controls for all experiments. Furthermore, as we have previously shown a gene dosage effect in mice with deletion of one or both alleles of *Rptor* in pre-osteoblasts^20^, heterozygous littermates were also included in all analyses.

**Figure 1:**
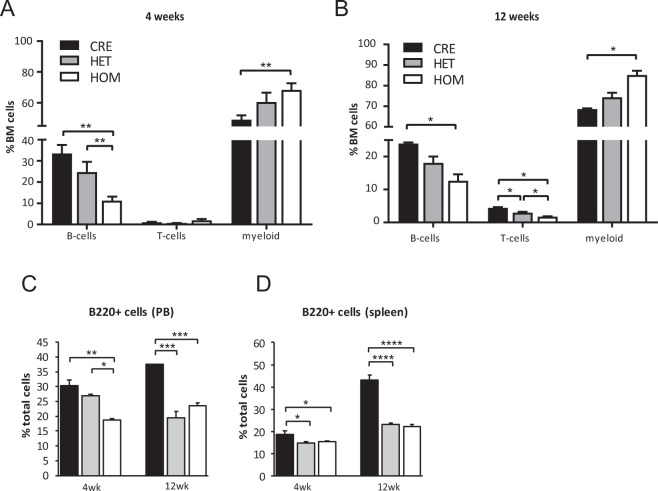
B-Lymphopoiesis is impaired in *eYFP-Rptor*_*ob*_^+/−^ *and eYFP-Rptor*_*ob*_^−/−^ mice at 4 and 12 weeks of age. The frequency of B-cells, T-cells and myeloid cells in the bone marrow (BM) of (**A**) 4-week old and (**B**) 12-week old *eYFP-Osx:Cre* (CRE), *eYFP-Rptor*_*ob*_^+/−^ (HET), and *eYFP-Rptor*_*ob*_^−/−^ (HOM) mice was assessed using flow cytometry (n = 4–5/group). Data are expressed as percentage of total bone marrow cells. (**C**) The percentage of B220^+^ B-lymphocytes in the peripheral blood (PB) of 4- and 12-week old mice (n = 4–5/group). (**D**) The percentage of B220^+^ B-lymphocytes in the spleens of 4- and 12-week old mice (n = 4–5/group). Data are expressed as mean ± SEM. *p < 0.05, **p < 0.01, ***p < 0.005, ****p < 0.001, one-way ANOVA with Tukey’s post-hoc test.

To determine whether the loss of *Rptor* affects the ability of osteoblasts to support haematopoietic development, we analysed the frequency of mature haematopoietic lineages in the BM of heterozygous (*eYFP-Rptor*_*ob*_^+/−^) and homozygous (*eYFP-Rptor*_*ob*_^−/−^) knockout mice using flow cytometry. For these studies, animals were analysed at both 4 and 12 weeks of age as they represent early and later phase of long-bone growth in mice^29^. As *eYFP-Rptor*_*ob*_^−/−^ mice are significantly smaller than *eYFP-Osx:Cre* controls at both 4 and 12 weeks of age^20^, the distribution of each lineage was calculated as a percentage of total BM cells in order to account for the reduced skeletal size and bone marrow cellularity of *eYFP-Rptor*_*ob*_^−/−^ mice. At both time points, a significant reduction in B220^+^ B-cells was observed in the BM of *eYFP-Rptor*_*ob*_^−/−^ knockout mice compared to *eYFP-Osx:Cre* controls (Fig. 1A,B). At 4 weeks of age, no significant difference in CD3^+^ T-cells was observed in the BM of *eYFP-Rptor*_*ob*_^−/−^ mice, whereas CD11b^+^/Gr1^+^ myeloid cells were increased (Fig. 1A). In contrast, a significant reduction in CD3^+^ T-cells and a significant increase in CD11b^+^/Gr1^+^ myeloid cells was observed in 12-week old *eYFP-Rptor*_*ob*_^−/−^ mice compared to controls (Fig. 1B). Notably, the B-cell defect observed in the BM of *eYFP-Rptor*_*ob*_^−/−^ knockout mice was accompanied by a significant reduction in the number of B220^+^ B-cells in the peripheral blood circulation (Fig. 1C) and the spleen (Fig. 1D) at both 4 and 12 weeks of age. A significant reduction in B220^+^ B-cells in the peripheral blood circulation and spleen of heterozygous *eYFP-Rptor*_*ob*_^+/−^ mice relative to controls was also observed at 12 weeks of age, suggestive of a gene dose effect (Fig. 1C,D).

**Figure 2:**
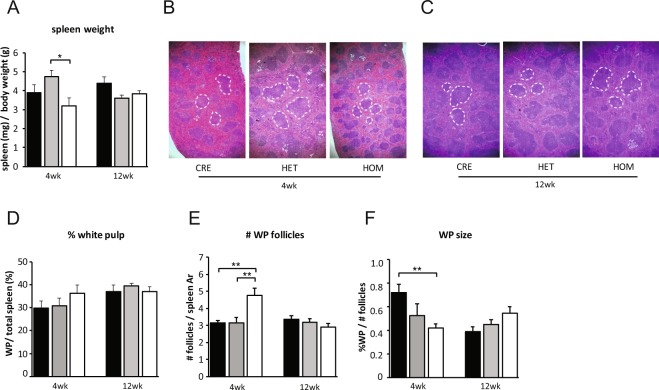
Loss of mTORC1 function is pre-osteoblasts affects spleen size and structure. (**A**) Spleen weights, normalised to total body weight, of 4- and 12-week old mice (n = 10–15/group). Representative haematoxylin and eosin-stained spleen sections from (**B**) 4-week old and (**C**) 12-week old *eYFP-Osx:Cre* (CRE), *eYFP-Rptor*_*ob*_^+/−^ (HET), and *eYFP-Rptor*_*ob*_^−/−^ (HOM) mice (4x magnification). The dotted white outlines indicate white pulp. (**D**) Quantitation of total white pulp area, expressed as a percentage of total spleen area, in 4- and 12-week old mice (n = 10/group). (**E**) Quantitation of white pulp follicle number, normalised to total spleen area analysed, in 4- and 12-week old mice (n = 10/group). (**F**) Quantitation of white pulp follicle size, in 4- and 12-week old mice (n = 10/group). Data are expressed as mean ± SEM. *p < 0.05, **p < 0.01, one-way ANOVA with Tukey’s post-hoc test.

Whilst the spleens of 4-week old *eYFP-Rptor*_*ob*_^−/−^ mice were markedly smaller than *eYFP-Osx:Cre* controls, this was not statistically significant (p = 0.64) when corrected for body weight (Fig. 2A). Intriguingly, *eYFP-Rptor*_*ob*_^−/−^ spleen weights were significantly reduced compared to the heterozygous *eYFP-Rptor*_*ob*_^+/−^ knockout mice, due to a modest increase in weight-adjusted spleen weight in the *eYFP-Rptor*_*ob*_^+/−^ mice compared to *eYFP-Osx:Cre* controls (Fig. 2A). Whilst *eYFP-Rptor*_*ob*_^+/−^ and *eYFP-Rptor*_*ob*_^−/−^ mice displayed reduced spleen weights compared to *eYFP-Osx:Cre* controls at 12 weeks of age, this was not statistically significant (p = 0.42 and p = 0.55 respectively, Fig. 2A). Within the spleen, the proliferation and differentiation of B-lymphocytes occurs in lymphoid follicles, the major component of the white pulp (Fig. 2B,C). While histological analysis revealed no difference in splenic white pulp area in *eYFP-Rptor*_*ob*_^+/−^ and *eYFP-Rptor*_*ob*_^−/−^ knockout mice compared to controls (Fig. 2D), the number of white pulp follicles was significantly increased in *eYFP-Rptor*_*ob*_^−/−^ at 4 weeks of age (Fig. 2E). As a result, the average size of the white pulp follicles in these mice was significantly reduced in 4-week old mice (Fig. 2F).

### B-Lymphopoiesis is Impaired Along the Pro-B to Pre-B Cell Transition in *eYFP-Rptor*_*ob*_^+/−^ and *eYFP-Rptor*_*ob*_^−/−^ Knockout Mice

**Figure 3:**
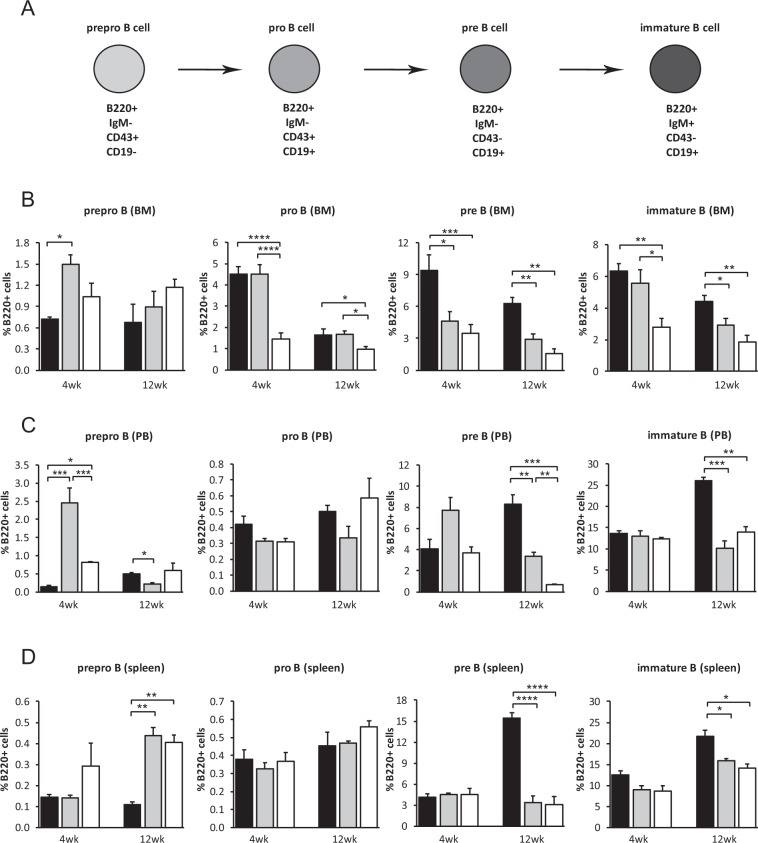
Impaired B-Lymphopoiesis in *eYFP-Rptor*_*ob*_^+/−^ *and eYFP-Rptor*_*ob*_^−/−^ mice is due to a stall in the Pro-B to Pre-B Cell Transition. (**A**) Immunophenotypic expression markers used to distinguish B220^+^ B cell subsets. Cells isolated from (**B**) the bone marrow (BM), (**C**) peripheral blood (PB) and (**D**) spleen of 4- and 12-week old *eYFP-Osx:Cre* (CRE), *eYFP-Rptor*_*ob*_^+/−^ (HET), and *eYFP-Rptor*_*ob*_^−/−^ (HOM) mice (n = 4–5/group) were stained with antibodies directed against the B-cell phenotypic markers CD19, CD43, IgM and B220. The number of prepro-B cells (B220^+^IgM^−^CD19^−^CD43^+^), pro-B cells (B220^+^IgM^−^CD19^+^CD43^+^), pre-B cells (B220^+^IgM^−^CD19^+^CD43^−^), and immature B-cells (B220^+^IgM^+^CD19^−^CD43^−^) present within each compartment was analysed using flow cytometry. Data are expressed as percentage of B220^+^ cells, mean ± SEM. *p < 0.05, **p < 0.01, ***p < 0.005, ****p < 0.001, one-way ANOVA with Tukey’s post-hoc test.

During B-lymphopoiesis, commitment of the common lymphoid progenitor to the B-cell lineage is marked by the expression of B220, followed by sequential differentiation to prepro-B, pro-B and pre-B cells and ultimately, immature B-cells. These immature B-cells then leave the BM for the final stages of their maturation in secondary lymphoid organs to form end-stage antibody-producing plasma cells which, upon activation by antigen-presentation, return and colonise the BM^7–12^. To investigate whether impaired B-lymphopoiesis is responsible for the reduced numbers of B220^+^ B-cells in *eYFP-Rptor*_*ob*_^+/−^ and *eYFP-Rptor*_*ob*_^−/−^ mice, the relative proportion of prepro-B, pro-B, pre-B and immature B-cells in the BM, spleen and peripheral blood circulation was assessed using CD19, CD43, IgM and B220 phenotypic markers (Fig. 3A; Supplementary Fig. 1). As shown in Fig. 3B, the percentage of pro-B cells, pre-B cells, and immature B-cells was significantly reduced in the BM of *eYFP-Rptor*_*ob*_^−/−^ mice at both 4 and 12 weeks of age. This reduction in pre-B and immature B cells was also observed in the peripheral blood circulation and the spleen of *eYFP-Rptor*_*ob*_^−/−^ mice albeit at 12 weeks of age only (Fig. 3C,D). Furthermore, this reduction was also evident in heterozygous *eYFP-Rptor*_*ob*_^+/−^ mice, consistent with a gene dose effect. Unlike the BM, there was no significant change in the number of pro-B cells in the peripheral blood or spleen irrespective of genotype or age (Fig. 3C,D). Interestingly, there was a significant increase in the number of prepro-B cells in the spleens of both heterozygous and homozygous mice, relative to controls, at 12 weeks of age (Fig. 3D). These data suggest that mTORC1 signalling in pre-osteoblasts has a profound effect on B-cell development.

### CXCL12 and IL-7 mRNA Expression is Decreased in *Rptor*-Deficient Osteoblasts

**Figure 4:**
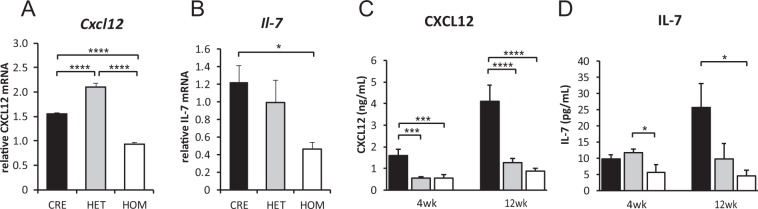
CXCL12 and IL-7 expression is decreased in *eYFP-Rptor*_*ob*_^+/−^ *and eYFP-Rptor*_*ob*_^−/−^ mice. (**A**,**B**) qPCR was performed on RNA extracted from eYFP^+^ osteoblast cells isolated from *eYFP-Osx:Cre* (CRE), *eYFP-Rptor*_*ob*_^+/−^ (HET), and *eYFP-Rptor*_*ob*_^−/−^ (HOM) mice. Levels of CXCL12 and IL-7 mRNA expression were measured, and data were normalised to β-actin. (**C**,**D**) Circulating levels of CXCL12 and IL-7 were measured in peripheral blood serum samples collected from 4- and 12-week old mice (n = 5–10/group) using commercial ELISA kits. *p < 0.05, ***p < 0.005, ****p < 0.001, one-way ANOVA with Tukey’s post-hoc test.

Skeletal osteoblasts are a rich source of trophic factors which regulate B-lymphopoiesis. CXCL12, an important regulator of early B-cell development, is important for prepro-B cell development^9^. In contrast, IL-7 is an important regulator of late B-cell development and is crucial for the production of pro-B and pre-B cells^30,31^. To identify the potential mechanisms by which pre-osteoblast mTORC1 signalling regulates B-lymphopoiesis, we assessed transcript levels of *Cxcl12* and *Il7* in eYFP^+^ cells (ie. osteoprogenitors, mature osteoblasts and osteocytes harbouring Cre-mediated recombination) recovered from the long bones of 4-week old *eYFP-Osx:Cre*, *eYFP-Rptor*_*ob*_^+/−^ and *eYFP-Rptor*_*ob*_^−/−^ mice. *Cxcl12* and *IL7* mRNA levels were significantly reduced in *eYFP-Rptor*_*ob*_^−/−^ osteoblasts compared to controls (Fig. 4A: mean decrease 39.5 ± 2.2%; and Fig. 4B: mean decrease 62 ± 6.1%). In contrast, for heterozygous *eYFP-Rptor*_*ob*_^+/−^ mice, transcript levels of *Cxcl12* were increased and no change in *Il7* transcript levels, relative to controls, was observed (Fig. 4A,B). Despite the genotype-specific differences in transcript levels a significant reduction in circulating CXCL12 levels was evident in 4- and 12-week old *eYFP-Rptor*_*ob*_^+/−^ and *eYFP-Rptor*_*ob*_^−/−^ mice compared to controls (Fig. 4C). Circulating IL-7 levels were significantly reduced in *eYFP-Rptor*_*ob*_^−/−^ mice compared to controls at 12 weeks of age only (Fig. 4D).

### *Rptor* deficient osteoblasts fail to support HSC differentiation to B-cells *in vitro*, but this can be rescued by exogenous Flt3L, IL-7 and CXCL12

To confirm that the abnormal B-lymphopoiesis observed in the *eYFP-Rptor*_*ob*_^−/−^ mice is directly attributable to *Rptor* deficiency in osteoblasts, we next examined the ability of wild type and *Rptor-*deficient osteoblasts to support the differentiation of purified haematopoietic stem cells (LSK cells) into B-cell precursors. To achieve this, primary calvarial MSCs were isolated from neonatal *Rptor*^*fl/fl*^ mice and infected with a tamoxifen-inducible self-deleting Cre recombinase (CreER^T2^). CreER^T2^-infected cells were then treated with or without tamoxifen for 8 days to induce *Rptor* deletion (RapKO) or vehicle control (WT) MSCs. These WT and RapKO MSCs were then cultured under osteoinductive conditions to produce RapKO and WT osteoblasts as previously described^6^.

**Figure 5:**
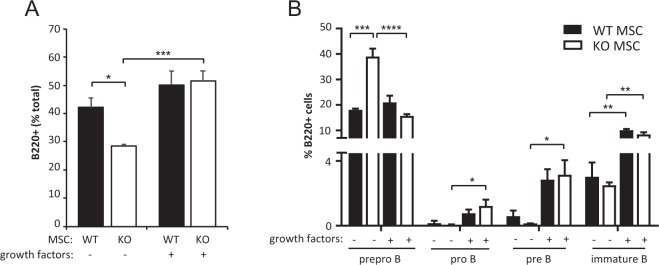
*Rptor* deficient osteoblasts are unable to support B-lymphopoiesis *in vitro* unless supplemented with exogenous CXCL12 and IL-7. The ability of wild type (WT) and *Rptor-*deficient osteoblasts (RapKO) to support B-lymphopoiesis *in vitro* was examined by co-culturing Lin^−^Sca-1^+^c-kit^+^ (LSK) cells on osteoblast monolayers in the presence or absence of exogenous growth factors. (**A**) The percentage of B220^+^ cells arising from *in vitro* co-culture was examined by flow cytometry. Data are expressed as a percentage of total haematopoietic cells. *p < 0.05, ***p < 0.005, one-way ANOVA with Tukey’s post-hoc test. (**B**) Haematopoietic cells recovered from WT and RapKO osteoblast co-cultures (in the presence or absence of exogenous growth factors) were stained with antibodies directed against the B-cell phenotypic markers CD19, CD43, IgM and B220. The number of prepro-B cells (B220^+^IgM^−^CD19^−^CD43^+^), pro-B cells (B220^+^IgM^−^CD19^+^CD43^+^), pre-B cells (B220^+^IgM^−^CD19^+^CD43^−^), and immature B-cells (B220^+^IgM^+^CD19^−^CD43^−^) was analysed using flow cytometry. Data are expressed as a percentage of B220^+^ cells, mean ± SEM. *p < 0.05, **p < 0.01, ***p < 0.005, ****p < 0.001, two-way ANOVA with Tukey’s multiple comparisons post-hoc test.

When BM LSK cells from wild type C57BL/6 mice were added to these osteoblast monolayers, approximately 42% of the haematopoietic cells recovered from the WT osteoblast co-cultures were B220^+^ after 10 days compared to only 29% of the cells recovered from RapKO osteoblast co-cultures (Fig. 5A: mean decrease 31.7 ± 1.5%). Importantly, the addition of exogenous IL-7 and CXCL12 to these co-cultures restored the ability of RapKO osteoblasts to support B lymphopoiesis, with 49% and 51% of the haematopoietic cells recovered from WT and RapKO osteoblast co-cultures found to be B220^+^, respectively (Fig. 5A).

Using CD19, CD43 and IgM phenotypic markers, the relative proportion of prepro-B, pro-B, pre-B and immature B-cells within the B220^+^ cells isolated from the osteoblast-LSK co-cultures was also examined. As shown in Fig. 5B, in the absence of exogenous factors, the percentage of prepro-B cells was significantly increased in RapKO osteoblast co-cultures compared to WT co-cultures (mean increase: 115.47 ± 17%), whereas the percentages of pro-B, pre-B and immature-B cells were reduced. Importantly, the addition of exogenous IL-7 and CXCL12 to these co-cultures restored the ability of RapKO osteoblasts to support LSK differentiation into pre-B and immature B-cells as evidenced by a factor-dependent normalisation of prepro-B cell numbers and a significant increase in the percentage of pro-B, pre-B and immature B cells (Fig. 5B).

## Discussion

Stromal cells within the BM microenvironment, such as osteoblasts, endothelial cells, adipocytes and fibroblasts, are crucial for HSC development. Beyond its support for HSC precursors, the BM stroma also provides a specific niche for lineage-committed haematopoietic cells such as B-lymphocytes^14^. Recent studies from our laboratory and others have demonstrated that mTORC1 signalling pathways play an important role in skeletal development, with bone-specific knockout of *mTOR*^32^ and *Rptor*^20–22^ leading to impaired postnatal bone acquisition and conversely, bone-specific knockout of tuberous sclerosis complex 2 (TSC-2, negative regulator of mTORC1)^32^ causing a progressive increase in postnatal bone acquisition. Considering the well-established role that osteoblasts play in supporting haematopoiesis and B-cell development^3,6^, we hypothesised that skeletal mTORC1 signalling plays an important role in supporting B-cell development.

In this study, we showed that the deletion of *Rptor* in Osterix-expressing cells has a profound effect on B-cell development, with a significant reduction in the number of pro-B, pre-B and immature B-cells observed in the BM of *eYFP-Rptor*_*ob*_^−/−^ mice compared to *eYFP-Osx:Cre* controls at both 4- and 12-weeks of age. This defect in B-cell development within the BM was accompanied by a reduction in the number of circulating pre-B and immature B cells in the blood. The substantial change in the proportion of B-cell precursors in our *eYFP-Rptor*_*ob*_^−/−^ mice was due to a stall in B-cell development between the pro-B to pre-B cell transition.

We also showed that eYFP^+^ bone cells isolated from *eYFP-Rptor*_*ob*_^−/−^ KO mice express significantly lower levels of *Cxcl12* and *Il-7* transcript compared to *eYFP-Osx:Cre* controls, and display a concomitant decrease in CXCL12 and IL-7 protein levels in the peripheral blood circulation. Given the well-established role of IL-7 in the differentiation of pro-B cells^30,31^, the reduced levels of IL-7 expression observed in osteoblasts isolated from *eYFP-Rptor*_*ob*_^−/−^ mice was consistent with the defect in B-lymphopoiesis at the pro-B to pre-B transition in these mice. The significant reduction in CXCL12 expression however, in the absence of any defect in prepro-B cell production in the BM, was somewhat surprising and raises the possibility that other factors may compensate for the loss of CXCL12 in the early stages of B-lymphopoiesis in these mice. CXCL12 is important for the formation of distinct niches in the bone marrow that support life-long generation of B lymphocytes by acting as a chemoattractant for CXCR4-expressing progenitors^33^. It is therefore interesting to note that the level of splenic prepro-B cells was elevated in 12-week old *eYFP-Rptor*_*ob*_^−/−^ mice. As these cells are typically restricted to the BM, an increase in splenic prepro-B cells could reflect a change to the B cell supportive niche that allows extravasation of progenitor cells to the spleen. Moreover, modification of bone marrow niche may also account for the reduction in T cells and increase in myeloid cells in *eYFP-Rptor*_*ob*_^−/−^ mice.

Whilst this manuscript was under preparation, a study by Wang and colleagues showed that the deletion of *Rptor* in *Osx*-expressing pre-osteoblasts, and not mature *Ocn-*expressing osteoblasts, leads to impaired B-lymphopoiesis^34^. In this study, the authors showed that 3-week-old homozygous *Osx-Rptor* KO mice display impaired B-cell differentiation and a block in B-cell development in the Pro-B to Pre-B transition, with this defect attributed to pre-B and immature B-cell apoptosis induced by abnormal IL-7/Stat5 signalling. A key difference between the Wang *et al*., study and this current study is the genotype of the control mice. We have compared *eYFP-Rptor*_*ob*_^−/−^ and *eYFP-Rptor*_*ob*_^+/−^ to *eYFP-Osx:Cre* mice (i.e. a control strain harbouring two wild type *Rptor* alleles), whereas Wang *et al*. compared the phenotype of homozygous *Rptor* KO mice to heterozygous *Rptor* KO mice. Given the well-documented skeletal phenotype of *tTA:Osx:Cre* mice^20,26–28^ and potential non-skeletal *Sp7* (Osterix) expression in the olfactory bulb and intestinal epithelia^35^, *eYFP-Osx:Cre* mice are an indispensible control for *Osx-Cre*-driven conditional gene deletion models. Indeed, as shown here, the observed perturbations in the BM, spleen and peripheral blood compartments of *eYFP-Rptor*_*ob*_^−/−^ mice were often magnified when compared to the *eYFP-Osx:Cre* control, and in some instances the inclusion of the *eYFP-Osx:Cre* control revealed differences that were not apparent when comparing to heterozygous KO animals alone.

Importantly, the comparison of *eYFP-Rptor*_*ob*_^−/−^ and *eYFP-Rptor*_*ob*_^+/−^ with *eYFP-Osx:Cre* control mice has enabled us to assess the effect of *Rptor* gene dose in pre-osteoblasts in the regulation of B-lymphopoiesis. As demonstrated herein, loss of one allele of *Rptor* in pre-osteoblasts is sufficient to cause significant alterations to the frequency and tissue distribution of B-cell subsets. This pattern of disruption does not mirror that observed in *eYFP-Rptor*_*ob*_^−/−^ mice, suggesting distinct cellular responses to the loss of one *vs*. two alleles. We observed a similar gene dose effect when comparing the skeletal phenotypes of *eYFP-Rptor*_*ob*_^+/−^ and *eYFP-Rptor*_*ob*_^−/−^ mice with *eYFP-Osx:*Cre mice, where deletion of one *Rptor* allele resulted in a less severe loss of bone mass and bone strength compared to deletion of both *Rptor* alleles^20^. Phenotypes arising from the reduction (loss of one allele of *Rptor*) or complete (homozygous deletion of *Rptor*) loss of mTORC1 function in pre-osteoblasts are complex as mTORC1 plays an important role in negative feedback loops that control signal transduction pathways. In response to insulin, mTORC1 directly and indirectly phosphorylates insulin receptor substrate 1 (IRS-1), leading to its degradation^36^. mTORC1 also activates growth factor receptor-bound protein 10 (Grb10), which inhibits IRS1 from binding to activated insulin receptor (InsR)^37^. Thus perturbations in mTORC1 function can lead to hyper-stimulation of signalling pathways which contribute to phenotype complexity.

We have previously shown that osteoblasts derived from *eYFP-Rptor*_*ob*_^−/−^ knockout mice have a reduced osteogenic potential and a transcriptional profile consistent with an immature osteoblast phenotype, indicating that *Rptor* deletion leads to a stall in early osteogenic differentiation^20^. These cells also have reduced protein synthetic capacity, which likely inhibits their capacity to differentiate into mature osteoblasts. Thus, we postulate that mTORC1-mediated translational control contributes to IL-7 and CXCL12 production in osteoblasts and is responsible for the impaired B-lymphopoiesis observed in response to osteoblastic mTORC1 inhibition.

In addition to playing an essential role in the maintenance and differentiation of HSCs, OBs have been shown to play a role in suppressing haematological malignancies. Osteoblast numbers are significantly decreased in patients with myelodysplasia and acute myeloid leukemia^38^, and genetic depletion of osteoblasts in murine models of acute leukemia results in an increase in circulating blasts and tumor engraftment^38^. Global disruption of gene expression by deletion of *Dicer1* in osteoprogenitors results in impaired osteoblastic differentiation and the development of myelodysplasia with the propensity to develop acute myeloid leukemia (AML)^39^. Similarly, constitutive activation of β-catenin signaling, specifically in osteoblast progenitors alters the differentiation potential of myeloid and lymphoid progenitors leading to the development of AML^40^. These findings demonstrate that critical changes in the signaling pathways that govern normal osteoblast function can contribute to aberrant haematopoiesis. Therefore, gaining a greater understanding of osteoblastic signaling pathways that are important for maintaining normal haematopoiesis could reveal important therapeutic targets for haematopoietic malignancies.

In summary, our findings demonstrate that mTORC1 signalling in osteoprogenitor cells is crucial to the regulation of B-lymphopoiesis by cells of the osteoblast lineage. Given the propensity for malignant disorders of the B-cell lineage (e.g. multiple myeloma) to involve the skeleton, these findings lay the foundation for future studies involving the pathophysiology of these disorders and offer novel therapeutic approaches.

## Materials and Methods

### Transgenic mice

All mice were bred and housed at the SA Pathology Animal Care Facility and all studies performed with Institutional Ethics approval (SA Pathology/CALHN, #102/12) and were performed in accordance with relevant guidelines and regulations. Conditional knockout mice in which *Rptor*, a unique and essential component of mTORC1, was disrupted in early osteoprogenitor cells were generated using *Osx1-GFP::Cre* mice^23^, R26eYFP mice^25^ and *Rptor*^*fl/fl*^ mice^24^ as previously described^20^. In all studies, *Osx1-GFP::Cre* (designated *eYFP-Osx:Cre*) mice were used as controls. Heterozygous (*Osx1-GFP::Cre-Rosa26eYFP-Rptor*^+/*fl*^, designated *eYFP-Rptor*_*ob*_^+/−^) and homozygous (*Osx1-GFP::Cre-Rosa26eYFP-Rptor*^*fl/fl*^, designated *eYFP-Rptor*_*ob*_^−/−^) knockout mice were born at the expected Mendelian frequencies and at birth, no gross phenotypic differences or difference in weight or length was evident in knockout animals, relative to age-matched *eYFP*-*Osx:Cre* controls^20^. For all studies, equal numbers of male and female mice were used in each group.

### Hematopoietic B-cell Staining and Flow Analysis

Bone marrow, spleen and peripheral blood cells were isolated from 4- and 12-week-old *eYFP-Osx:Cre*, *eYFP-Rptor*_*ob*_^+/−^ and *eYFP-Rptor*_*ob*_^−/−^ mice (n = 5–8 per group). Peripheral blood was haemolysed by osmotic lysis using ammonium chloride buffer (150 mM NH_4_Cl, 10 mM KHCO_3_, 0.1 mM EDTA, pH 7.3) on ice for 10 minutes. BM, spleen and peripheral blood cells were stained with antibodies to B-lymphocytes (B220, IgM), T-lymphocytes (CD3e) and granulocytes (CD11b, Gr-1). B-cell precursors were analysed with antibodies to CD19-APC (clone 1D3, eBioscience, CA, USA), CD43-PE (clone 2/60, eBioscience), IgM-PE-Cy7 (clone R6-60.2, BD Bioscience, NJ, USA), B220-biotin (clone RA3-6B2, BioLegend, CA, USA) and streptavidin-FITC (Southern Biotech, AL, USA). Flow cytometric analysis was performed on a BD LSR Fortessa X20 Analyser and analysed using FlowJo software.

### Immunohistochemistry

Sections (4 μm) of formalin-fixed paraffin-embedded spleens isolated from 4- and 12-week-old *eYFP-Osx:Cre*, *eYFP-Rptor*_*ob*_^+/−^ and *eYFP-Rptor*_*ob*_^−/−^ mice (n = 8–10 per group) were stained with haematoxylin and eosin. Histomorphometric analysis of white pulp area, total spleen area and follicle number was performed using the OsteoMeasure XP system (Osteometrics Inc., GA, USA). For quantitative assessment of white pulp area, follicle number and follicle size between mouse cohorts, values were normalised to total spleen area in order to account for the reduced spleen size of *eYFP-Rptor*_*ob*_^−/−^ mice.

### Isolation of eYFP^+^ cells and real-time PCR

Compact bone cells (CB-MSCs) were obtained from the long bones of 4-week-old *eYFP-Osx:Cre*, *eYFP-Rptor*_*ob*_^+/−^ and *eYFP-Raptor*_*ob*_^−/−^ mice in accordance to published methods^41^. In brief, femora and tibiae from 8–12 mice were excised, cleaned thoroughly then crushed in ice-cold phosphate buffered saline (PBS) supplemented with 2% FCS and 2 mM EDTA, washed several times, chopped into small fragments and digested in 3 mg/mL Type 1 collagenase (Worthington Biochemical Corporation, NJ, USA) at 37 °C for 45 minutes with shaking. The resulting cell suspension was filtered through a 70 μm strainer then eYFP^+^ cells isolated by FACS using a MoFlo® Astrios™ (Beckman Coulter, CA, USA). Total RNA was isolated from eYFP^+^ cells using the RNAqueous-Micro kit (Life Technologies, Melbourne, Australia) and cDNA prepared from 70 ng of total RNA using Sensiscript reverse transcriptase as per the manufacturers’ instructions (Qiagen). Real-time PCR reactions were performed using RT^2^ SYBR Green ROX reagent (Qiagen) in Rotor-Gene 3000 thermo-cycler (Corbett Research, Qiagen). Primer sets used in this study: *β-actin*: Fwd 5′-ttgctgacaggatgcagaag-3′; Rev 5′-aagggtgtaaaacgcagctc-3′, *Cxcl12*: Fwd 5′-gtcctcttgctgtccagctc-3′; Rev 5′-gtctgttgttgttcttcagcc-3′, *Il-7*: Fwd 5′-attatgggtggtgagagccg-3′; Rev 5′-gttcctgtcattttgtccaattca-3′. Changes in gene expression were calculated relative to β-actin using the 2^−∆∆Ct^ method^42^.

### ELISA

Circulating levels of CXCL12 and IL-7 were measured in serum samples collected from 4- and 12-week-old animals using commercial murine-specific immunoassay kits as per manufacturer’s instructions (R&D Systems, MN, USA).

### Calvarial MSC Isolation

Primary calvarial MSCs were isolated from 4-day old *RosaRaptor*^*fl/fl*^ mice as previously described^43^. Briefly, calvariae excised from 4 litters of mice were pooled, washed with PBS and digested in 2 mg/mL Type 2 collagenase at 37 °C (Worthington Biochemical Corporation) for 20 minutes on a shaking platform. The resultant cell suspension was pelleted by centrifugation. Fresh collagenase was added to the calvariae for an additional 20 minutes and the resultant cell suspension was pooled with the cells from the first round of digestion.

Calvarial MSCs were cultured in α-MEM supplemented with 20% FCS, 2 mM L-glutamine, 100 µM L-ascorbate-2-phosphate, 50 IU/mL penicillin, 50 μg/mL streptomycin sulphate, 1 mM sodium pyruvate and 15 mM HEPES (Sigma Aldrich, NSW, Australia) in a hypoxic *in vitro* cabinet (5% O_2_, 10% CO_2_, 85% N_2,_ Coy Laboratory Products, MI, USA) at 37 °C^41^. Cells were passaged by detachment with a 0.05% trypsin-EDTA solution upon reaching 80–90% confluence.

### Cre-mediated gene deletion

Calvarial MSCs (passages 2–3) were infected with a lentivirus carrying a tamoxifen-inducible self-deleting Cre recombinase (LEGO-CreER^T2^-iG2)^44^ as previously described^45^. Calvarial MSCs with stable lentiviral integration were selected on the basis of GFP expression. Following *in vitro* expansion, cells were treated with 0.5 μM 4-hydroxytamoxifen (4-OHT, Sigma Aldrich) for 8 days to induce *Rptor* deletion (RapKO cells). Ethanol (0.05%) was used as a vehicle control.

### Osteoblast/haematopoietic cell co-culture

Osteoblastic differentiation of calvarial MSCs was induced by the addition of 10 mM β-glycerol phosphate (Sigma), 50 μM ascorbate-2-phosphate, 10^−7^ M dexamethasone and 100 ng/mL human BMP-2 in 10% FCS/α-MEM for 5–6 days until the cells reached confluence, followed by digestion and replating in the presence of 10 mM β-glycerol phosphate, 50 μM ascorbate-2-phosphate and 10^−7^ M rat PTH (Sigma Aldrich) for 7–10 days, until the cells reached confluence and formed a gel-like collagen pad^6^.

BM Lin^-^Sca-1^+^c-kit^+^ (LSK) cells from wild type mice were used as a source of primary HSCs. Briefly, whole BM cells were flushed from the femurs and tibias, blocked with mouse γ-globulin (Jackson Immunoresearch) and stained with a cocktail of biotinylated antibodies to mature haematopoietic cell markers (CD3, CD4, CD5, CD8, CD11b, Gr-1, B220, Ter-119; BioLegend), Sca1-PE-Cy7 (BD Bioscience), c-kit APC (eBioscience), and fluorogold (Abcam, Cambridge, UK) to exclude non-viable cells. Fluorescence Activated Cell Sorting (FACS) was performed to collect LSK cells (FACSAriaIII, Becton Dickinson).

LSK cells (1000 cells/well) were seeded onto monolayers of pre-cultured osteoblasts with or without cytokines (30 ng/mL mIL-7, 30 ng/mL mFlt-3L and 200 ng/mL mCXCL12, R&D Systems) in 10% FCS/α-MEM containing 50 μM ascorbate-2-phosphate and 10 mM β-glycerol phosphate, and co-cultured for 10 days. On day 5, media was changed and fresh IL-7 and Flt-3L added to cytokine-treated wells. After 10 days, cells were stained using antibodies directed against B220, IgM, CD43 and CD19 (as described above) and analysed on a BD LSR Fortessa X20 Analyser.

### Statistical analysis

All data are presented as mean ± standard error of the mean (SEM). Statistical significance was determined by one-way or two-way ANOVA with Tukey’s post-hoc test using GraphPad Prism (Version 6.0; GraphPad Software Inc, CA, USA). In all cases, p < 0.05 was considered statistically significant.

## Electronic supplementary material

Supplementary Information
