# Supplementary material for: mTORC1 plays an important role in osteoblastic regulation of B-lymphopoiesis
Source: Sci Rep. 2018 Sep 28;8:14501. doi: 10.1038/s41598-018-32858-5 (PMC6162303; doi:10.1038/s41598-018-32858-5)

mTORC1 plays an important role in osteoblastic regulation of B-lymphopoiesis

Sally K Martin[1,2], Stephen Fitter[1,2], Nadia El Khawanky[2,3,4], Randall H Grose[2], Carl R Walkley[5], Louise E Purton[5], Markus A Ruegg[6], Michael N Hall[6], Stan Gronthos[2,7], Andrew CW Zannettino[1,2*].

[1]Myeloma Research Laboratory, Adelaide Medical School, Faculty of Health and Medical Sciences, University of Adelaide, Adelaide, Australia.
[2]The South Australian Health and Medical Research Institute, Adelaide, Australia.
[3]School of Medicine, Faculty of Health and Medical Sciences, University of Adelaide, Adelaide, Australia.
[4]Department of Hematology and Oncology, Faculty of Medicine, University of Freiburg, Freiburg, Germany.
[5]Stem Cell Regulation Unit, St Vincent's Institute of Medical Research, Melbourne, Australia.
[6]Biozentrum, University of Basel, Basel, Switzerland.
[7]Mesenchymal Stem Cell Laboratory, Adelaide Medical School, Faculty of Health and Medical Sciences, University of Adelaide, Adelaide, Australia.

SUPPLEMENTARY FIGURE 1. The flow cytometric gating strategy used to identify B cell subset populations. The relative proportion of prepro-B, pro-B, pre-B and immature B-cells in the BM, spleen and peripheral blood circulation was assessed using CD19, CD43, IgM and B220 phenotypic markers.

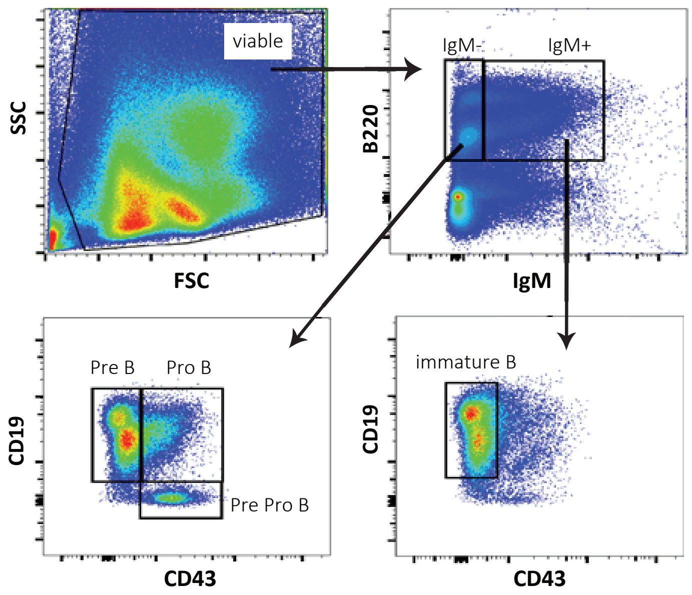

Supplement: Supplementary file 1 — Supplementary Information [file 41598_2018_32858_MOESM1_ESM.pdf]
